# Dysprosium Acetylacetonato Single-Molecule Magnet Encapsulated in Carbon Nanotubes

**DOI:** 10.3390/ma10010007

**Published:** 2016-12-23

**Authors:** Ryo Nakanishi, Mudasir Ahmad Yatoo, Keiichi Katoh, Brian K. Breedlove, Masahiro Yamashita

**Affiliations:** 1 Department of Chemistry, Graduate School of Science, Tohoku University, 6-3 Aza-Aoba, Aoba-ku, Sendai, Miyagi 980-8578, Japan; muda.amu@gmail.com (M.A.Y.); kkatoh@m.tohoku.ac.jp (K.K.); breedlove@m.tohoku.ac.jp (B.K.B.); 2 WPI Research Center, Advanced Institute for Materials Research, Tohoku University, 2-1-1 Katahira, Aoba-ku, Sendai 980-8577, Japan

**Keywords:** single-molecule magnet, carbon nanotube

## Abstract

Dy single-molecule magnets (SMMs), which have several potential uses in a variety of applications, such as quantum computing, were encapsulated in multi-walled carbon nanotubes (MWCNTs) by using a capillary method. Encapsulation was confirmed by using transmission electron microscopy (TEM). In alternating current magnetic measurements, the magnetic susceptibilities of the Dy acetylacetonato complexes showed clear frequency dependence even inside the MWCNTs, meaning that this hybrid can be used as magnetic materials in devices.

## 1. Introduction

Single-molecule magnets (SMMs) [1,2,3,4], which are composed of isolated molecules, usually with large spin angular momenta (*S*) in the ground state and strong uniaxial magnetic anisotropies (*D*), exhibit an extensive range of functional properties, like magnetic bistability [1], quantum tunneling of magnetization [5,6,7,8], and quantum coherence [9]. Thus, they can be considered as not only molecular equivalents of classical bulk ferromagnets but also as next-generation quantum magnets. Therefore, SMMs are being developed for application in memory storage and in the processing of quantum information [10,11]. Moreover, novel applications of SMMs, including their use in molecular spintronics [12] and quantum computing [13], are being explored.

To use SMMs, we must be able to exploit the functionality of individual SMM molecules and combine them with other functional materials. There have been a few reports on combining SMMs with materials. For example, SMMs have been combined with carbon nanotubes (CNTs) [14] and graphene [15]. From these examples, when lanthanoid SMMs interact with nanocarbon materials, their electronic properties are affected. Another example involves the encapsulation of SMMs into nanoscopic one-dimensional pores, such as the internal nano-space of CNTs [16] and metal-organic frameworks [17], in which SMMs become aligned and their magnetic properties are controlled. SMM-nanomaterial hybrids may have new structures and unique physical properties. If SMMs are encapsulated in one-dimensional pores, the stacking structure can be controlled, and the SMM properties should be enhanced. Furthermore, when SMMs are encapsulated in CNTs, they are protected from the surrounding environment, and thus, the hybrids are easier to use in real applications. However, little has been reported on lanthanoid SMMs encapsulated inside CNTs. In this work, we encapsulated Dy acetylacetonato SMMs [18] in multi-walled CNTs (MWCNTs) by using a capillary method [19,20]. Encapsulation was verified by using transmission electron microscopy (TEM). It was shown that Dy complexes maintained their SMM-like properties in the MWCNTs.

## 2. Results and Discussion

### 2.1. Synthesis

MWCNTs with an internal diameter of ~5 nm were purified by using centrifugation [21], and then the end-caps were opened by heating in air. The impurities in the internal nano-space were removed by heating in a vacuum. Next, Dy(acac)_3_(H_2_O)_2_ was dissolved in 1,2-dichloroethane, and the solution was heated at 65 °C for 2 h to obtain a saturated solution. Cap-opened MWCNTs were added to the saturated solution and dispersed by using ultrasonication. Then the solution was left to stand for 3 d in order to encapsulate Dy(acac)_3_(H_2_O)_2_ into the MWCNTs via a capillary phenomenon [19,20]. After filtering and washing the surfaces with 1,2-dichloroethane, Dy(acac)_3_(H_2_O)_2_ encapsulated in MWCNTs (Dy(acac)_3_(H_2_O)_2_@MWCNTs) were obtained.

### 2.2. Transmission Electron Microscopy, Elemental Analysis and Thermogravimetry

**Figure 1:**
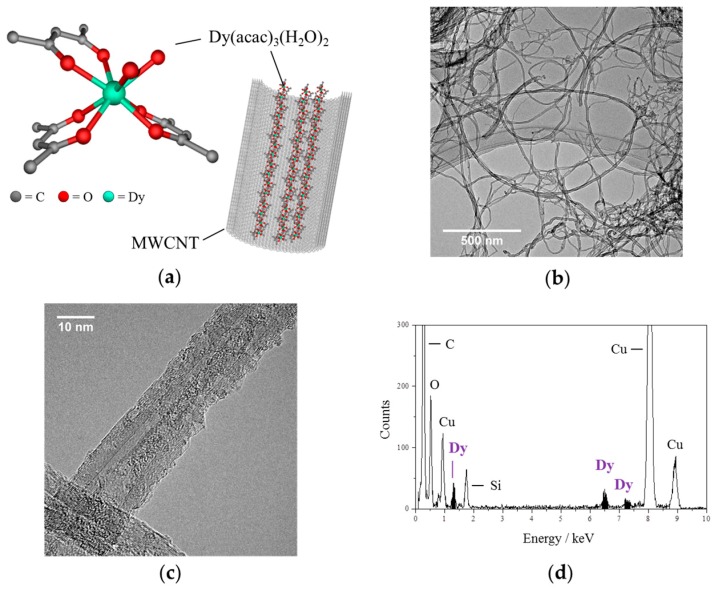
(**a**) Drawings of Dy(acac)_3_(H_2_O)_2_ complex and the complexes encapsulated in multi-walled carbon nanotubes (MWCNT); (**b**) Low magnification and (**c**) high magnification transmission electron microscopy (TEM) images of Dy(acac)_3_(H_2_O)_2_@MWCNTs; (**d**) energy dispersive X-ray spectroscopy (EDX) spectrum acquired for the sample in (**c**).

**Figure 2:**
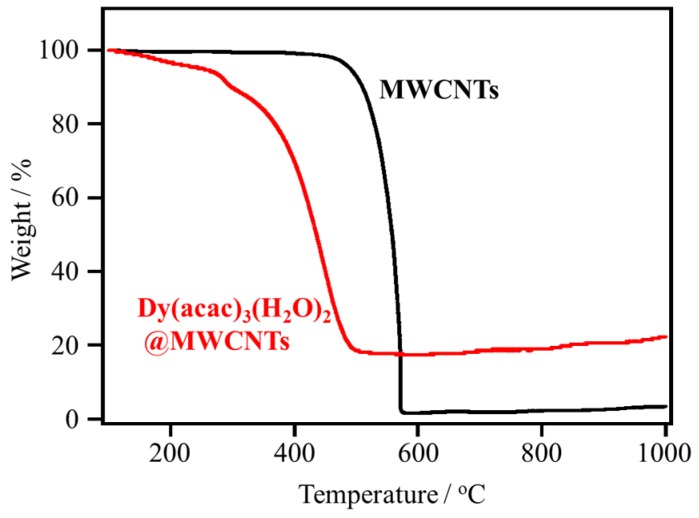
Thermogravimetric analyses of empty MWCNTs (black) and Dy(acac)_3_(H_2_O)_2_@MWCNTs (red).

TEM was used to view the interior of the MWCNT hybrids; the structure images are illustrated in Figure 1a. In the TEM images, only Dy(acac)_3_(H_2_O)_2_@MWCNTs as free-standing entities were observed, and there were no complexes on the external surfaces of the MWCNTs (Figure 1b). In enlarged images, a stark contrast between the Dy(acac)_3_(H_2_O)_2_@MWCNT (Figure 1c) and the empty MWCNTs was observed, as shown in Supplementary Materials Figure S1, showing that Dy(acac)_3_(H_2_O)_2_ was encapsulated. In order to confirm the encapsulation and characterize the material present inside the MWCNTs, energy-dispersive X-ray (EDX) spectroscopy was used to detect the Dy ions (Figure 1d). The results clearly indicate that Dy(acac)_3_(H_2_O)_2_ is encapsulated in the MWCNTs. Thermogravimetric analysis (TGA) was performed on pristine MWCNTs and Dy(acac)_3_(H_2_O)_2_@MWCNT (Figure 2). For the pristine MWCNTs, when *T* > 600 °C, all of the carbon was lost as CO_2_. However, in the case of Dy(acac)_3_(H_2_O)_2_@MWCNT, 22.3 wt % of a white compound remained even when *T* > 1000 °C. This material is thought to be Dy_2_O_3_. From the TGA data, the amount of Dy(acac)_3_(H_2_O)_2_ was estimated to be 1.2 mmol in 1 g of Dy(acac)_3_(H_2_O)_2_@MWCNT.

### 2.3. Magnetic Properties

**Figure 3:**
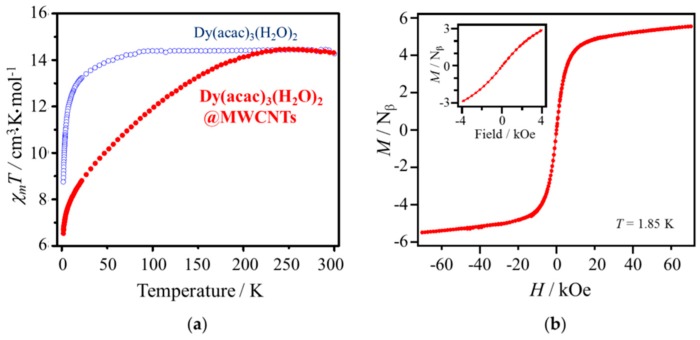
(**a**) *χ*_m_*T* vs. *T* plots for Dy(acac)_3_(H_2_O)_2_@MWCNTs (red filled circles) and pure Dy(acac)_3_(H_2_O)_2_ (blue open circles); (**b**) *M* vs. *H* plots for Dy(acac)_3_(H_2_O)_2_@MWCNTs at 1.85 K. The inset shows magnified curve in the range of −4–4 kOe.

To determine the effects of encapsulation of the SMMs in MWCNTs on the magnetic properties, both static and dynamic magnetic measurements on Dy(acac)_3_(H_2_O)_2_@MWCNTs were performed, and the results were compared with those for free Dy complexes. Direct current (DC) measurements were used to obtain molar magnetic susceptibilities (*χ*_m_), which depended on *T* and the magnetic field (*H*). *χ*_m_*T*-*T* plots for Dy(acac)_3_(H_2_O)_2_@MWCNTs and pure Dy(acac)_3_(H_2_O)_2_ are shown in Figure 3a. After correcting the diamagnetism of the MWCNTs (see Supplementary Materials Figure S2), we determined the *χ*_m_ values for Dy(acac)_3_(H_2_O)_2_@MWCNTs by using the ratio obtained from TGA, and the resulting *χ*_m_*T* value at 300 K agrees with that for an isolated Dy(III) ion (14.2 cm^3^·K·mol^−1^), which suggests that the estimated amount of Dy(acac)_3_(H_2_O)_2_ is reliable. *χ*_m_*T* values for Dy(acac)_3_(H_2_O)_2_@MWCNTs decreased with a decrease in *T*, whereas those for pure Dy(acac)_3_(H_2_O)_2_ did not. This difference was ascribed to depopulation of high energy *m*_J_ states due to configurational and orientational changes in the ligands upon encapsulation [22,23].

In magnetization (*M*) vs. *H* plots, shown in Figure 3b, magnetic hysteresis was not observed. In the case of Dy(acac)_3_(H_2_O)_2_ diluted with 20 equivalents of Y(acac)_3_(H_2_O)_2_, slight hysteresis has been observed at 2 K because the distance between each Dy(acac)_3_(H_2_O)_2_ is large and quantum tunneling of the magnetization (QTM) is suppressed [18]. Therefore, QTM is not suppressed for the Dy(acac)_3_(H_2_O)_2_@MWCNTs. In addition, it is possible that the coordination environment of Dy(acac)_3_(H_2_O)_2_ changed upon encapsulation in the MWCNTs, which promotes the QTM process and shortens the relaxation time. Similar behavior for Mn_12_-acetate SMMs encapsulated in MWCNTs has been reported [16]. In other words, no hysteresis was observed for the Dy hybrids. Thus, by controlling the coordination environment via encapsulation in CNTs, the relaxation time of the SMMs can be tuned.

**Figure 4:**
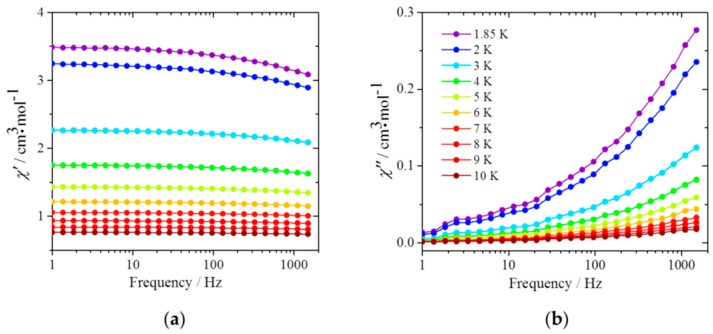
Frequency dependence of the (**a**) in-phase (*χ*′) and (**b**) out-of-phase (*χ*″) AC magnetic susceptibilities of Dy(acac)_3_(H_2_O)_2_@MWCNTs. The measurements were performed in an *H*_DC_ of 0 Oe and *H*_AC_ of 3 Oe in the *T* range of 1.85–10 K. The solid lines are guides for eyes.

**Figure 5:**
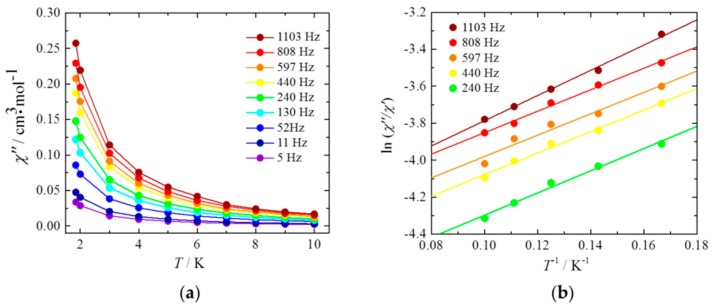
(**a**) *χ*″ vs. T plots for Dy(acac)_3_(H_2_O)_2_@MWCNTs. The solid lines are guides for eyes; (**b**) *χ*″/*χ*′ versus *T*^−1^ (6–10 K) plot in the *ν* range of 240–1103 Hz. The solid lines were fitted as described in Supplementary Materials Table S1.

Next, the dynamic magnetic properties were studied, and the results are shown in Figure 4. For Dy(acac)_3_(H_2_O)_2_@MWCNTs, an out-of-phase (*χ*″) signal, which is indicative of slow relaxation of *M*, was observed. Furthermore, both the in-phase (*χ*′) and *χ*″ signals were frequency dependent. This dependence is due to the Dy(acac)_3_(H_2_O)_2_ complexes because the susceptibilities of the MWCNTs themselves are not frequency dependent (Supplementary Materials Figure S3). These results indicate that the observed slow relaxation is due to SMM behavior, that is, there is an energy barrier for relaxation of the magnetic moment even inside the MWCNTs. However, there was no peak top for the Dy(acac)_3_(H_2_O)_2_@MWCNTs in the frequency range of 1–1000 Hz, whereas a clear peak top was observed for the pure complex (Supplementary Materials Figure S4). As seen in Figure 4b, peak top values of *χ*″ shifted towards higher frequencies. This indicates that the relaxation times for the hybrids are faster than those for the pure complex. In the *χ*″ versus *T* plots shown in Figure 5a, a peak top was still observed in the *T* region below 2 K, indicating that the magnetic moment was not frozen and that a different relaxation process, like QTM process, was dominant in the low-*T* region. We estimated the pre-exponential factor *τ*_0_ and the activation energy Δ*E* from *χ*″/*χ*′ versus *T*^−1^ (6–10 K) plots, shown in Figure 5b, in the *ν* range of 240–1103 Hz by using the Kramers-Kronig equation [23,24,25,26,27]:

(1) $$\chi^{\prime \prime }/\chi^{\prime }=\omega \tau$$

(2) $$\chi^{\prime \prime }/\chi^{\prime }=\omega \tau_{0}+\exp\;(∆E/k_{B}T)$$

(3) $$\ln\left(\chi^{\prime \prime }/\chi^{\prime }\right)=\ln\left(\omega \tau_{0}\right)+∆E/k_{B}T$$

where *ω* (=2*πν*) is the angular frequency. By fitting the data, the *τ*_0_ and Δ*E* for Dy(acac)_3_(H_2_O)_2_@MWCNTs were estimated to be in the range of 10^−6^–10^−7^ s and 4–5 cm^−1^, respectively (Supplementary Materials Table S1). For pure Dy(acac)_3_(H_2_O)_2_, *τ*_0_ and Δ*E* were determined to be 8.0 × 10^−7^ s and 45.9 cm^−1^, respectively [18]. We think that Δ*E* for the hybrids is lower because of a conformational change in Dy(acac)_3_(H_2_O)_2_ inside the MWCNTs. The values are consistent with the decrease in the *χ*_m_*T* value and magnetic hysteresis behavior.

## 3. Materials and Methods

### 3.1. General

Distilled water was obtained from a EYELA STILL ACE SA-2100E deionizer (Tokyo Rikakikai Co., Ltd., Tokyo, Japan). Dy(acac)_3_(H_2_O)_2_ (STREM Chemicals, Inc., Newburyport, MA, USA), 1,2-dichloroethane and methanol (Wako Pure Chemical Industries, Ltd., Osaka, Japan) were used as received. MWCNTs synthesized by using the CoMoCAT™ catalytic chemical vapor deposition method with outer diameters of 10 ± 0.1 nm, inner diameters of 4.5 ± 0.5 nm, and lengths of 3–6 µm (Sigma-Aldrich Co. LLC., St. Louis, MO, USA) were purchased and used after removing the magnetic impurities by using a centrifugation method [21]. The MWCNTs (30 mg) were dispersed with 60 mL of 1 wt % sodium cholate in water by using ultrasonication with a tip-type sonicator (UP200S, Hielscher Ultrasonics GmbH, Teltow, Germany). The obtained black suspension was centrifuged at 18,500 rpm for ~1 h using a tabletop centrifuge (AS185, AS ONE Co., Osaka, Japan), and the upper 80% of the supernatant was collected. The well-dispersed MWCNTs were aggregated by adding methanol and filtered over a Kiriyama filter (Kiriyama glass Co., Tokyo, Japan) having a pore size of 1 µm. The aggregates were then washed with excess methanol and dried at 200 °C in a vacuum overnight, affording 15 mg of purified MWCNT buckypaper.

### 3.2. Synthesis

Purified MWCNTs were decapped by heating at 550 °C for 5 min in air and degassed by heating in a vacuum just before using. To a saturated solution of Dy(acac)_3_(H_2_O)_2_ in 10 mL of 1,2-dichloroethane, which was heated at 65 °C for about 2 h to ensure that Dy(acac)_3_(H_2_O)_2_ dissolved as much as possible, 10 mg of decapped MWCNTs were added. After 5 min of ultrasonication using a bath-type sonicator and letting stand for 3 d, MWCNTs were collected by filtration and washed with 1,2-dichloroethane to completely remove the Dy(acac)_3_(H_2_O)_2_ from the surfaces of the MWCNTs.

### 3.3. TEM Observation

High-resolution transmission electron microscopy (TEM) and energy dispersive X-ray spectroscopy (EDX) were carried out using a JEM2100F (acceleration voltage; 200 kV, JEOL Ltd., Tokyo, Japan) with dry SD30GV detector (JEOL Ltd., Tokyo, Japan). The sample was dispersed in methanol and deposited on a carbon-coated Cu grid, which was dried by heating overnight at 100 °C in a 10^−4^ Pa vacuum before TEM was performed.

### 3.4. Thermogravimetric Analysis

Thermogravimetric analysis (TGA) was performed on a SHIMADZU DTG-60 (Shimadzu Corporation, Kyoto, Japan) using aluminum oxide powder as a standard material. Several milligrams of the sample were put in an aluminum cell, and the cell was heated to 1000 °C with a heating rate of 2 °C/min.

### 3.5. Magnetic Susceptibility Measurement

Magnetic susceptibility measurements were performed on a SQUID magnetometer (model MPMS-XL SQUID magnetometer, Quantum Design, Inc., San Diego, CA, USA). Samples were put into gelatin capsules, and eicosane was added to fix the samples during the measurement. DC measurements for Dy(acac)_3_(H_2_O)_2_ were performed in an *H*_DC_ of 500 Oe, and those for the purified MWCNTs and Dy(acac)_3_(H_2_O)_2_@MWCNTs were recorded in *H*_DC_ of 1000 Oe. *T* was changed from 300 K to 1.85 K with a sweep rate of 1 K/min. Field dependent DC measurements were performed at 1.85 K while changing the magnetic field as follows: 0 Oe → 70 kOe → −70 kOe → 70 kOe. AC measurements were recorded in an *H*_AC_ of 3 Oe in the frequency range of 1–1500 Hz and *T* range of 1.85–10 K. Diamagnetic contributions from the eicosane and Dy(acac)_3_(H_2_O)_2_ were corrected by using Pascal’s constants, and then the magnetic susceptibility for the purified MWCNTs was subtracted from that for Dy(acac)_3_(H_2_O)_2_@MWCNTs. Magnetic moments *χ*_CNT_, *χ*_CNT_′ and *χ*_CNT_″ (Supplementary Materials Figures S2 and S3) were obtained by normalizing the obtained magnetic moments with the mass of CNT after applying the diamagnetic corrections.

## 4. Conclusions

In this work, we encapsulated Dy(acac)_3_(H_2_O)_2_ SMMs in the internal nanospace of MWCNTs by using a capillary method. Encapsulation was confirmed by using TEM. From AC magnetic susceptibility measurements, both the in-phase and out-of-phase signals were clearly frequency dependent, indicating that Dy(acac)_3_(H_2_O)_2_ complexes still exhibited SMM-like properties. To the best of our knowledge, this is the first example of a lanthanoid SMM encapsulated in CNTs. Although the encapsulation of Dy(acac)_3_(H_2_O)_2_ into MWCNTs did not enhance the SMM properties, this work shows that it is possible to control the coordination environment and tune the magnetic properties of SMMs via encapsulation. In addition, we believe that the magnetic and electronic properties of lanthanoid SMM-CNT hybrids can be combined to bring about new applications in devices, like spintronic devices.

## Supplementary Materials

The following are available online at www.mdpi.com/1996-1944/10/1/7/s1. Figure S1: TEM image and EDX spectrum of empty MWCNT, Figure S2: *χ*_CNT_ and *χ*_CNT_*T* vs. *T* plots for MWCNT and Dy(acac)_3_(H_2_O)_2_@MWCNTs without correction for the diamagnetism of the MWCNTs, Figure S3: Temperature-dependence of the in-phase (*χ*′) and out-of-phase (*χ*″) AC magnetic susceptibilities of MWCNT and Dy(acac)_3_(H_2_O)_2_@MWCNTs, Figure S4: Frequency-dependence of *χ*′ and *χ*″ AC magnetic susceptibilities of Dy(acac)_3_(H_2_O)_2_, Table S1: Selected values of ∆*E* and $\tau_{0}$ for Dy(acac)_3_(H_2_O)_2_@MWCNTs.
