# Supplementary material for: Dysprosium Acetylacetonato Single-Molecule Magnet Encapsulated in Carbon Nanotubes
Source: Materials (Basel). 2016 Dec 23;10(1):7. doi: 10.3390/ma10010007 (PMC5344606; doi:10.3390/ma10010007)
Supplement: Supplementary file 1 [file materials-10-00007-s001.pdf]

Supplementary Materials: Dysprosium Acetylacetonato Single-Molecule Magnet Encapsulated in Carbon Nanotubes

Ryo Nakanishi, Mudasir Ahmad Yatoo, Keiichi Katoh, Brian K. Breedlove and Masahiro Yamashita

Table S1. Selected values of ΔE and τ_0 for $Dy(acac)_3(H_2O)_2$@MWCNTs estimated from the Kramers-Kronig equation.

ν/Hz	**240**	**440**	**597**	**808**	**1102**
ΔE/cm^{-1}	4.2	4.0	4.0	4.0	4.7
τ_0/s	5.0×10^{-6}	3.4×10^{-6}	2.8×10^{-6}	2.3×10^{-6}	1.7×10^{-6}

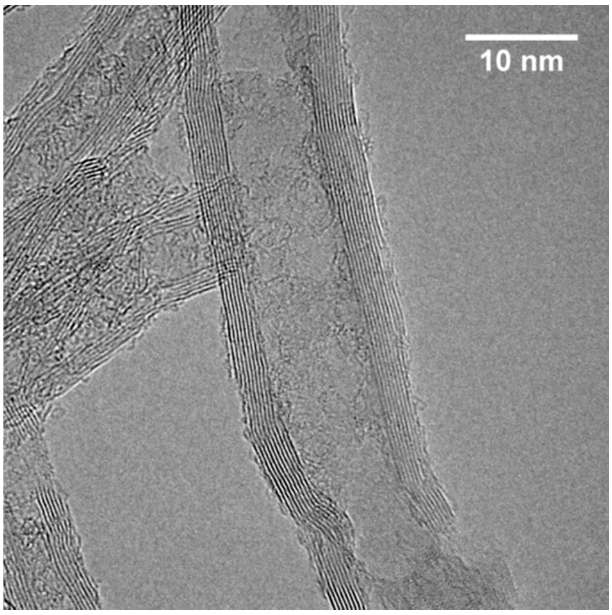

(**a**)

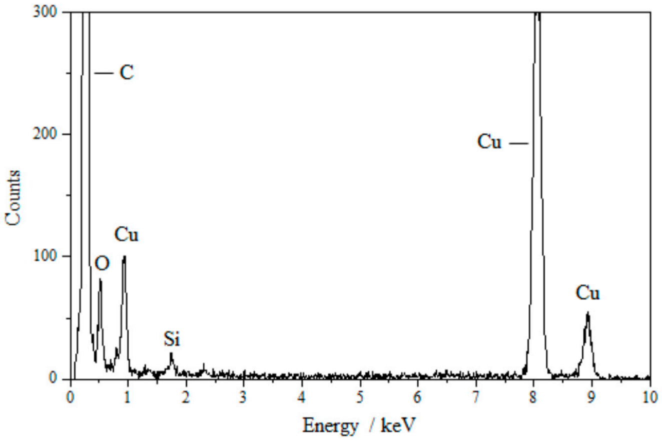

(**b**)

Figure S1. (**a**) TEM image of empty MWCNT; (**b**) EDX spectrum acquired for the sample in (**a**).

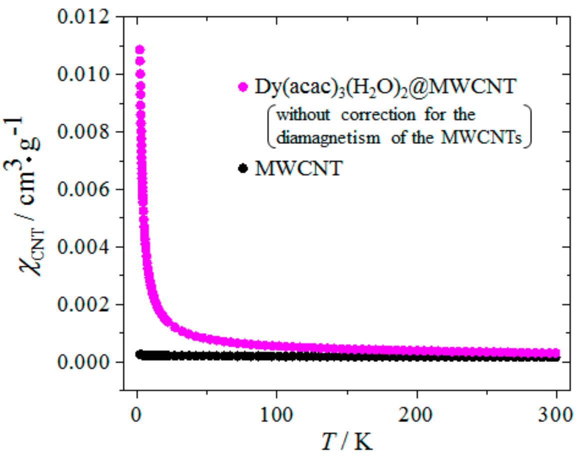

(**a**)

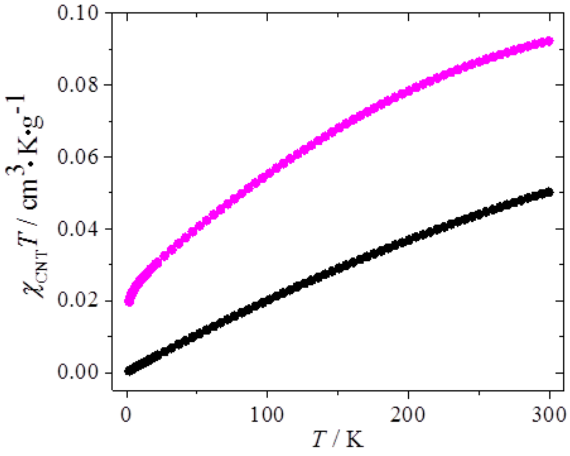

(**b**)

Figure S2. (**a**) χ_{CNT} and (**b**) $\chi_{CNT}T$ vs. T plots for MWCNT (black filled circles) and $Dy(acac)_3(H_2O)_2$@MWCNTs without correction for the diamagnetism of the MWCNTs (pink circles). χ_{CNT} values were obtained by normalizing the obtained magnetic moment with the mass of CNT after applying the diamagnetic corrections using Pascal's constants.

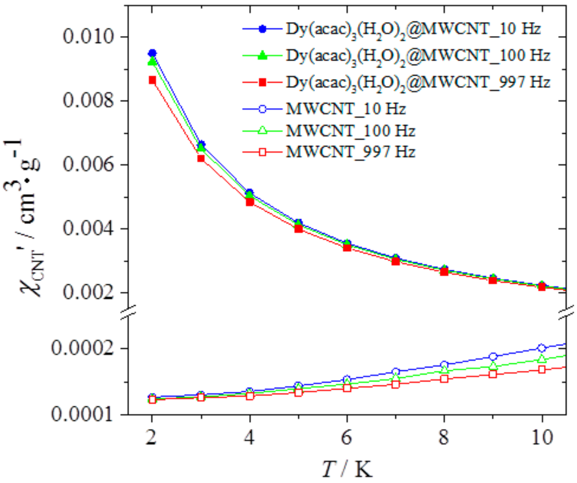

(**a**)

Figure S3. *Cont.*

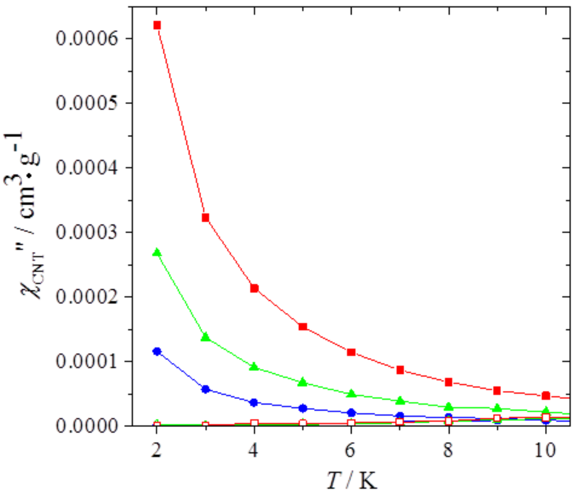

(**b**)

Figure S3. (**a**) χ_{CNT}' and (**b**) χ_{CNT}'' vs. *T* for $Dy(acac)_3(H_2O)_2$@MWCNT (filled symbols) and MWCNT (open squares) in an H_{DC} of 0 Oe. The measurements were performed in an H_{AC} of 3 Oe and *T* range of 10–2 K. χ_{CNT}' and χ_{CNT}'' were obtained by normalizing the obtained magnetic moment with the mass of CNT after applying the diamagnetic corrections using Pascal's constants.

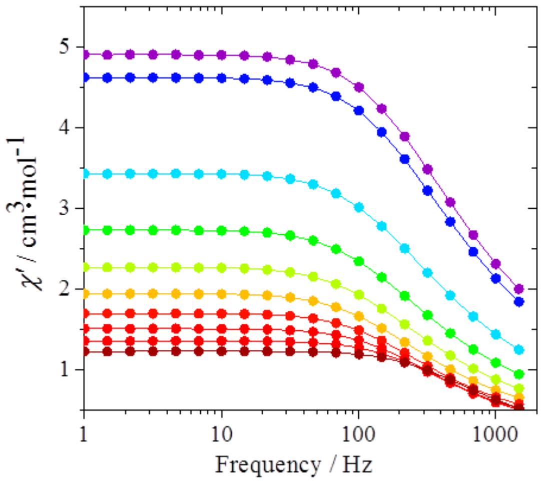

(**a**)

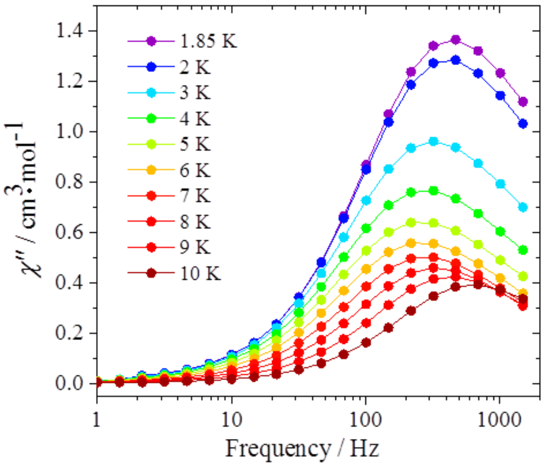

(**b**)

Figure S4. (**a**) χ' and (**b**) χ'' vs. frequency plots for $Dy(acac)_3(H_2O)_2$ in an H_{DC} of 0 Oe. The measurements were performed in an H_{AC} of 3 Oe and *T* range of 10–1.85 K. The solid lines are guides for eyes.
